# Characteristics of Bed Bug Infested Patients in the Emergency Department

**DOI:** 10.1155/2019/8721829

**Published:** 2019-05-09

**Authors:** Johnathan M. Sheele, Cameron J. Crandall, Brandon F. Chang, Brianna L. Arko, Colin T. Dunn, Alejandro Negrete

**Affiliations:** ^1^Department of Emergency Medicine, University Hospitals Cleveland Medical Center, 11100 Euclid Ave., B-517, Cleveland, OH 44139, USA; ^2^Case Western Reserve University School of Medicine, Cleveland, OH 44139, USA; ^3^Kent State University, Kent, OH, USA

## Abstract

*Cimex lectularius* L., the common bed bug, is a hematophagous human ectoparasite that has undergone a global resurgence in the past two decades. We surveyed 706 active emergency department (ED) patients about their experiences with bed bugs. We found that 2% of ED patients reported having a current bed bug infestation, significantly more than the historical number of ED patients upon which we find bed bug; 37% of ED patients report previously having been fed on by a bed bug; 15% currently know someone with an active infestation; and 59% know someone that has had an infestation within ≤ 5 years. Only 18% of bed bug infested patients reported their infestation to emergency medicine providers and only 21% were put in isolation precautions. We found that 25% of patients with bed bugs worried about receiving worse healthcare because of their infestation. Persons with bed bugs were more likely compared to those without bed bugs to be older (52 vs. 41 years) and arrive by ambulance (57% vs. 14%) (p < 0.05), but not reporting insomnia (50% vs. 49%) (p = 1.0). Bed bug infested patients can be common in the ED. Most bed bug infested patients are older, arrive to the ED by ambulance, do not report their infestation to healthcare providers, and are not adequately placed into isolation precautions, potentially putting other patients and providers at risk for acquiring the infestation.

## 1. Introduction

*Cimex lectularius* L., known as the common bed bug, is one of the most frequent parasites encountered by emergency medicine physicians in industrialized nations [1–5]. After near extirpation, in the past two decades the number of bed bug infestations has risen dramatically in many parts of the world including the United States [6, 7]. There has not been a systematic evaluation of* C. lectularius* as a vector of human disease, but the insect is associated with urticarial rashes and several psychiatric concerns including anxiety, depression, and insomnia [6–9].

Very little is known about the epidemiology of bed bugs in the emergency department (ED). For instance, the number of bed bug introductions in the ED is unknown and likely underreported partly because healthcare workers predominantly only find the larger insects, and no International Statistical Classification of Diseases and Related Health Problems (ICD-10) code exists for bed bug infested patients to study the problem [1, 2]. It is unclear if older and sicker ED patients are more likely to have a home bed bug infestation as well as the degree to which the insect can contribute to excess morbidity [3]. The objective of our study was to estimate the prevalence of bed bug infested patients in an ED and determine how infested patients were presenting to the ED and how often infested patients were being placed in isolation.

## 2. Methods

We received Institutional Review Board (IRB) approval to survey 706 ED patients in a single center, academic, tertiary care, level 1 trauma center in Cleveland, OH, between June and October 2017. All patients were ≥18 years of age and none had dementia, psychosis, homicidal ideation, or altered mentation. Data was collected seven days a week and predominantly during the day and evening hours. We estimate that we surveyed ~2-3% of all ED patients during our data collection. Beds bug isolation precautions at our institution involve putting a patient in a single occupancy room with the door closed, the patient's clothing and belongings closed inside two plastic bags, a bed sheet placed across the bottom doorway to hopefully prevent bed bugs from leaving the room, and the patient being placed on contact precautions. A professional pest management professional cleans the ED room after the patient leaves the ED. Chi-square, unpaired t-tests, and comparison of proportions were used in the analysis.

## 3. Results

We found that 2% (14/698) of patients reported a current home bed bug infestation, with five patients reporting “no answer” and three patients reporting “unsure”. This approximates to ~3-4 bed bug infested patients being seen in our ED daily. The average age for our patients' with bed bugs was significantly higher than those without bed bugs, 52 years (standard deviation (SD) 14) versus 41 years (SD 18; n = 692) (p < 0.02), respectively. Additionally, 37% (253/680) of patients report having been previously fed upon by a bed bug (25 unsure and 1 no answer), 59% (415/702) knew someone—other than themselves and people that live with them—that had a bed bug infestation in the past five years (4 unsure), and 15% (106/700) knew someone—other than themselves or someone that lives with them—that currently has a home bed bug infestation (6 unsure). The data is summarized in Table 1.

Patients with reported home bed bug infestations were significantly more likely than those without bed bug infestations to arrive to the ED by ambulance (57% versus 14%; p < 0.05). All patients that were placed into bed bug isolation precautions either came to the ED by ambulance 67% (4/6) or were dropped off by someone that they do not live with 33% (2/6). This contrasts with 14% of those without bed bugs (n = 684) that came by ambulance and 12% that were dropped off by someone they do not live with.

Only 21% (3/14) of those reporting a home bed bug infestation were placed in bed bug isolation precautions at the time the survey was completed compared to 0.4% (3/684) of persons reporting no active home bed bug infestation (3 unsure and 5 no answers) (p < 0.001). Those reporting no home infestation but were in ED bed bug isolation precautions indicate that ED staff found a bed bug on or next to the person suggesting the patient either did not know they had a home infestation, the subject did not respond truthfully to the survey question, or the bed bug was already in the healthcare environment prior to the patients' arrival. Interestingly, 64% (7/11) patients that reported home bed bugs stated they felt “no chance” (on a scale of 1-5 with 1 being “no chance” and 5 being “very likely”) that they could have brought bed bugs with them to the ED.

Only 18% (2/11) of persons with bed bugs notified EMS providers or ED staff about their home infestation. The reasons for people not reporting their home bed bug infestations to EMS or ED staff were that they either “did not think about it”, “did not think it was necessary”, “were not asked”, “did not feel like it was a problem”, felt like they were in "too much distress", and felt like it was "too embarrassing". Additionally, 25% (2/8) reported a concern that notifying ED staff that they had bed bugs would result in them receiving inferior care in the hospital (1 unsure, 5 no answers).

We found that 73% (8/11) of patients with bed bugs reported being embarrassed about having bed bugs, and 27% (3/11) reported having seen their primary care physician for help in managing their bed bug infestations. We found that 77% (10/13) of patients' with bed bugs were currently trying to eliminate their home infestations, but that only 55% (6/11) were using a pest management professional.

There were no significant differences (p > 0.05) between those that reported home bed bug infestations and those without infestations for the following: report trouble sleeping at night (50% versus 49%), reports someone that lives with them uses a wheelchair or walker (14% versus 12%), or having visitors with the person in the ED (14% versus 31%).

## 4. Discussion

We conducted the first large prospective survey of ED patients to better understand the prevalence of bed bugs in an ED. The survey took place in Cleveland, OH, one of the most bed bug infested areas in the United States, and it shows that bed bug infestations are a significant problem for many of our ED patients [10]. The hospital in which the survey was performed previously reported finding a bed bug in the institution approximately every 2 days, with most insects found in the ED at a frequency of every 3-5 days [2, 5]. The direct annual costs for the ED to decontaminate a patient room due to a bed bug were approximately $50,000 per year [5]. Our current survey suggests that our ED treats at least a 10-folder greater number of bed bug infested patients than we are identifying by just finding the insect on patients.

A survey of patients that had a bed bug on or near them in the ED found that these patients were more likely to be male, older in age, have higher emergency severity index (ESI) scores, be admitted to the hospital, and arrive to the ED by ambulance [2, 3]. Our findings are consistent with previous research showing that patients with bed bugs were more likely to be older and arrive by ambulance than uninfested patients. It remains unclear if persons with bed bugs are at baseline more likely to be chronically ill, whether they have fewer resources (e.g., needed transportation to the ED), or whether the bed bugs are indirectly or directly contributing to human disease.

Most bed bug infested patients did not report their infestation to EMS providers and were not placed into isolation precautions in the ED. This represents a missed opportunity for the healthcare system to intervene on behalf of the patient as well as a failure to correctly triage infested patients using isolation precautions to prevent the insects from infesting other patients and healthcare providers.

## 5. Limitations

Our survey represents a convenience sample of ~2-3% of all ED patients over the course of five months, and all surveys were from a single center. The low number of patients reporting bed bugs limits the generalizability. If possible, the additional data obtained from the critically ill, intoxicated, and mentally ill ED patients would have provided a more accurate estimate of ED patients with bed bugs. Previous research has suggested that ED patients with bed bugs are more likely to be older, sicker, and associated with several psychiatric conditions common in ED patients such as anxiety and depression which could result in an underreporting of bed bug infestations in our ED [3, 6–8]. We were unable to confirm the presence of bed bug infestations in the majority of patients. However, a door-to-door survey in Philadelphia found the reported prevalence of bed bugs to be 11%, and infestation was able to be confirmed in 68% of cases which indicates that persons are able to accurately self-report the presence of home bed bug infestations [11].

## 6. Conclusions

Our results support previous work suggesting that the number of persons with bed bugs in the ED is significantly underreported [1]. In our survey, 2% of ED patients reported being infested with bed bugs, 37% report that they have previously been fed on by a bed bug, and 18% know someone that currently has a bed bug infestation. Persons reporting home bed bug infestations are more likely to be older and arrive to the ED by ambulance; unlikely be put into bed bug isolation precautions in the ED; report that bed bugs cause them significant distress; not disclose to EMS providers or ED staff that they have bed bugs at home; feel embarrassed about having bed bugs; and feel it is unlike that they brought the insects with them to the ED.

Not all persons with a home bed bug infestation will bring the insects with them to the ED and having busy ED staff screening the belongings and clothing for everyone reporting home bed bug infestations may not be practical as the insects can be small, reclusive, and difficult to identify. The feasibility of putting everyone with a reported home bed bug infestations on bug isolation precautions remains unknown, but having an infested patient bringing bed bugs into the ED puts other patients and ED staff at risk for acquiring an infestation. Emergency medicine policies and procedures should be developed to appropriately identify and intervene with patients with bed bugs to help prevent the spread of the infestation.

## Figures and Tables

**Table 1:** Survey results comparing those with and without an active home bed bug infestation.

	+ current home bed bug infestation (n = 14)	- current home bed bug infestation (n = 684)	p-value
Placed in bed bug isolation precautions at the time of data collection in the ED	Yes: 3 (21%)	Yes: 3 (0.4%)	< 0.0001
	No: 11 (79%)	No: 681 (99.6%)	

Do any of your neighbors currently have bed bugs?	Yes: 4 (40%)	Yes: 35 (6%)	p < 0.001
	No: 6 (60%)	No: 548 (94%)	
	(Unsure: 1	(Unsure: 101	
	No answer: 3)	No answer: 22)	

Mechanism of ED arrival	Walked: 7%	Walked: 7%	
	Bus: 0%	Bus: 12%	
	Dropped off by someone you live with: 21%	Dropped off by someone you live with: 26%	
	Dropped off by someone you don't live with: 0%	Dropped off by someone you don't live with: 12%	
	Police: 0%	Police: 0.5%	
	Ambulance: 57%	Ambulance: 14%	
	Drove self: 14%	Drove self: 28%	
	Other: 0%	Other: 0.5%	

Do you have trouble sleeping at night?	Yes: 7 (50%)	Yes: 329 (49%)	1.0
	No: 7 (50%)	No: 350 (51%)	
		Unsure: 2	
		No answer: 3	

Does anyone that lives primarily with you use a wheelchair or a walker?	Yes: 2 (14%)	Yes: 83 (12%)	0.69
	No: 12 (86%)	No: 593 (88%)	
		No answer: 8	

Do you think bed bugs transmit infectious diseases to humans?	Yes: 6 (43%)	Yes: 396 (58%)	0.5
	No: 3 (21%)	No: 94 (14%)	
	Unsure: 5 (36%)	Unsure: 193 (28%)	
		No answer: 1	

Do you have visitors with you today in the ED?	Yes: 2 (14%)	Yes: 209 (31%)	0.25
	No: 12 (86%)	No: 474 (69%)	
		No answer: 1	

Currently embarrassed about having bed bugs at home	Yes: 8 (73%)	N/A	
	No: 3 (27%)		
	No answer: 3		

Have you been to your primary care doctor to get help treating your bed bugs	Yes: 3 (27%)	N/A	
	No: 8 (73%)		
	No answer: 3		

How likely do you think it is that you brought bed bugs into the ED today (0 = no chance to 5 = very likely)	5=Very likely: 4 (36%)	N/A	
	4= (0%)		
	3=(0%)		
	2= (0%)		
	1= No chance: 7 (64%)		
	No answer: 3		

Did you alert EMS and/or ED staff that you have bed bugs at home?	Yes: 2 (18%)	N/A	
	No: 9 (82%)		
	No answer: 3		

If you didn't alert ED staff that you had bed bugs are you concerned that by doing so you might receive worse care in the hospital	Yes: 2 (25%)	N/A	
	No: 6 (75%)		
	No answer: 3		

If you didn't alert ED staff that you had bed bugs are you concerned that others might get bed bugs from you	Yes: 4 (57%)	N/A	
	No: 3 (43%)		
	Unsure: 2		

How much distress do bed bugs cause you at home	Lots: 5 (50%)	N/A	
	Moderate: 2 (20%)		
	Little: 2 (20%)		
	None: 1 (10%)		
	No answer: 4		

Are you currently trying to get rid of your bed bugs?	Yes: 10 (77%)	N/A	
	No: 3 (23%)		
	No answer: 1		

## Data Availability

The data used to support the findings of this study are included within the article.

## References

[1] Sheele J. M., Barrett E., Dash D., Ridge G. E. (2017). Analysis of the life stages of Cimex lectularius captured within a medical centre suggests that the true numbers of bed bug introductions are under-reported. *Journal of Hospital Infection*.

[2] Sheele J. M., Barrett E., Farhan O., Morris N. (2017). Analysis of bed bug (cimex lectularius) introductions into an academic medical center. *Infection Control and Hospital Epidemiology*.

[3] Sheele J. M., Gaines S., Maurer N. (2017). A survey of patients with bed bugs in the emergency department. *The American Journal of Emergency Medicine*.

[4] Langley R., Mack K., Haileyesus T., Proescholdbell S., Annest J. L. (2014). National estimates of noncanine bite and sting injuries treated in US hospital emergency departments, 2001-2010. *Wilderness & Environmental Medicine*.

[5] Totten V., Charbonneau H., Hoch W., Shah S., Sheele J. M. (2016). The cost of decontaminating an ED after finding a bed bug: Results from a single academic medical center. *The American Journal of Emergency Medicine*.

[6] Goddard J., DeShazo R. (2009). Bed bugs (cimex lectularius) and clinical consequences of their bites. *Journal of the American Medical Association*.

[7] Doggett S. L., Dwyer D. E., Peñas P. F., Russell R. C. (2012). Bed bugs: Clinical relevance and control options. *Clinical Microbiology Reviews*.

[8] Susser S. R., Perron S., Fournier M. (2012). Mental health effects from urban bed bug infestation (Cimex lectularius L.): A cross-sectional study. *BMJ Open*.

[9] Minocha R., Wang C., Dang K., Webb C. E., Fernández-Peñas P., Doggett S. L. (2017). Systemic and erythrodermic reactions following repeated exposure to bites from the Common bed bug Cimex lectularius (Hemiptera: Cimicidae). *Austral Entomology*.

[10] Orkin Orkin releases top 50 bed bug cities list. https://www.orkin.com/press-room/orkin-releases-top-50-bed-bug-cities-list.

[11] Wu Y., Tracy D. M., Barbarin A. M., Barbu C. M., Levy M. Z. (2014). A door-to-door survey of bed bug (Cimex lectularius) infestations in row homes in Philadelphia, Pennsylvania. *The American Journal of Tropical Medicine and Hygiene*.

